# Effects of Nonstarch Genetic Modifications on Starch Structure and Properties

**DOI:** 10.3390/foods9020222

**Published:** 2020-02-20

**Authors:** Shiyao Yu, Dengxiang Du, Alex C. Wu, Yeming Bai, Peng Wu, Cheng Li, Robert G. Gilbert

**Affiliations:** 1 Joint International Research Laboratory of Agriculture and Agri-Product Safety of Ministry of Education of China, Yangzhou University, Yangzhou 225009, China; shiyao.yu@uq.net.au (S.Y.); yeming.bai@uqconnect.edu.au (Y.B.); Licheng@usst.edu.cn (C.L.); 2 The University of Queensland, Centre for Nutrition and Food Science, Queensland Alliance for Agriculture and Food Innovation, Brisbane, QLD 4072, Australia; wupengxmu@163.com; 3 National Key Laboratory of Crop Genetic Improvement, Huazhong Agriculture University, Wuhan 430030, China; dudengxiang@webmail.hzau.edu.cn; 4 The University of Queensland, Centre for Crop Science, Queensland Alliance for Agriculture and Food Innovation, Brisbane, QLD 4072, Australia; c.wu1@uq.edu.au; 5 College of Chemistry and Chemical Engineering, Xiamen University, Fujian Province 361005, China; 6 School of Medical Instrument and Food Engineering, University of Shanghai for Science and Technology, Shanghai 200093, China

**Keywords:** starch, molecular structure, transgenic, digestibility, gelatinization

## Abstract

This paper examines if, in maize, starch structure and starch-dependent properties might be altered by pleiotropic effects arising from genetic modifications that are not directly related to starch synthesis. The molecular structure, specifically the starch chain-length distributions (CLDs), of two maize lines transformed with *Bar* (*bialaphos resistance*) and *Cry1c* genes (an artificial gene, encoding proteinaceous insecticidal δ-endotoxins) were compared to those of their control lines. The two transgenes are responsible for herbicidal resistance and insect tolerance, respectively. The starch CLDs were measured by enzymatic debranching and measuring the molecular weight distributions of the resulting linear chains. It was found that although all the lines had similar amylose contents, the CLDs of both amylopectin and amylose for *Cry1c* were noticeably different from the others, having more short amylopectin and long amylose chains. These CLDs are known to affect functional properties, and indeed it was found that the *Cry1c* transgenic lines showed a lower gelatinization temperature and faster digestion rate than the control or *Bar* lines. However, a slower digestion rate is nutritionally desirable. Thus, pleiotropic effects from genetic modifications can indirectly but significantly affect the starch synthesis pathway and thus change functional properties of significance for human health.

## 1. Introduction

Genetic engineering is an avenue to improve crop traits such as yield, stress tolerance, and nutrition. Many studies have focused on phenotypic effects (changes in characteristics resulting from interactions between the organism’s genetics and its environment) of the exogenous (introduced) genes, but pleiotropic effects (unanticipated changes arising from the introduced genes) have only occasionally been noted [1,2,3,4]. Pleiotropic effects on starch molecular structure can be of importance, because this structure is a major determinant of functional properties of starch produced by the crop [5]. As it is difficult to characterize changes of starch molecular structures via field observations, pleiotropic effects on starch quality have been neglected. 

Starch, the main component of cereal endosperm, is the largest single energy source in human food. There are two types of starch molecules, amylose and amylopectin, both with (1→4)-α linear links and (1→6)-α branch points. Amylopectin is a large molecule with a vast number of short branches, while amylose is somewhat smaller, with a few long-chain branches. Amylopectin chains in native starch form double helices with each other in crystalline lamellae, while amylose is located in the amorphous lamellae which form alternating layers in starch granules. Starch molecular structure has a close relation to starch properties, such as gelatinization and digestibility (e.g., [5]). The amylopectin chain-length distribution (CLD, the distribution of the number or weight of monomer units in individual branches) is largely responsible for gelatinization characteristics [6]. Amylose content is also important for many functional properties. However, these are not the only controlling factors; for example, amylose molecular size and CLD have been found to be strongly correlated to rheological and gelatinization properties for starches with similar amylose content [7]. The fine structures of amylose and amylopectin have been shown to have an important role in starch digestibility, especially for gelatinized starch (e.g., [7]).

This study focuses on possible changes in starch structure arising from transformation of two genes which do not have a direct role in starch biosynthesis. These are transformations which are widely used in genetic modifications: a pest-resistant gene (*Cry1c* gene) and a herbicide-resistant gene (*bialaphos* resistance gene, *Bar* gene). The transformation of the *Bar, USA.* gene has been applied in soybean, maize, cotton, wheat, and canola [8]. There have been no investigations as to whether either of these transgenes can lead to a change in starch structure. Although it was reported [9] that the starch from three transgenic rice lines with the *Cry1Ab* gene (an artificially engineered *Bt* gene) had different viscosities from the wild type, starch molecular structures were not examined in that study.

In the present study, starch molecular structures resulting from *Cry1c* and *Bar* mutations are characterized using size-exclusion chromatography (SEC, a type of gel-permeation chromatography, GPC) to find the weight distributions of individual chains (following enzymatic debranching). The debranched distributions, the CLDs, are fitted with two mathematical models, one for amylopectin biosynthesis and one for amylose biosynthesis, to obtain a set of biologically meaningful parameters related to starch biosynthesis that quantify differences in CLDs from the various lines. These parameterizations closely fit the CLD over the chosen fitting range (e.g., [5]) and the fitting parameters can then be used to find statistically acceptable relations and differences between the structural characteristics for different varieties. We also examine two types of starch properties which are strongly influenced by the starch CLD: thermal properties (involving gelatinization) and digestibility after cooking and retrogradation.

## 2. Materials and Methods

### 2.1. Materials

Three varieties of transgenic maize grains (*bar*, *Cry1c*, and *control*, denoting herbicide-resistant lines with the *Bar* gene, pest resistant lines with the *Cry1c* gene, and control lines, respectively), fully matured and each with two bioreplicas, were kindly provided by Dr Dengxiang Du from Huazhong Agriculture University, Wuhan, China. All the transgenic maize grains were harvested from the same experimental field of Huazhong Agriculture University (Wuhan, China) at the same time. All were harvested from the fifth generation of three transformed and untransformed maize lines, cultivated from Hi-II embryogenic callus culture. The plasmid construction, transformation process, and plant cultivation information have been fully described elsewhere [10]. T1 was in 2014, and the samples for this work were harvested in 2017.

Protease from *Streptomyces griseus* (type XIV), pancreatin (P1750) from porcine pancreas, and LiBr (ReagentPlus) were purchased from Sigma-Aldrich Pty. Ltd. (Castle Hill, Australia). Isoamylase, amyloglucosidase from *Pseudomona* sp., D-Glucose (GOPOD Format) Assay Kit, and a series of pullulan standards with peak molecular weights ranging from 342 to 2.35 × 10^6^ Da were purchased from Megazyme International, Ltd. (Bray, Co. Wicklow, Ireland) and Polymer Standards Service (PSS) (Mainz, Germany), respectively. Dimethyl sulfoxide [DMSO, guaranteed reagent (GR) grade for analysis] was purchased from Merck KGaA, Darmstadt, Germany. All other chemicals were reagent-grade and used as received.

### 2.2. Maize Grain Starch Purification

The maize grains were frozen in liquid nitrogen before being cryoground into powder with a mixer mill (MM 400, Retsch GmbH, Haan, Germany) for 2 min at a frequency of 15 s^–1^. The finely ground powder was purified with a wet milling procedure following a method described elsewhere [11]. Each cryoground sample (5 g) was steeped in sodium bisulfite solution (30 mL, 0.45%, *w*/*v*) at 4 ℃ overnight and then homogenized in sodium metabisulfite solution using a homogenizer (T-25 Ultra-Turrax, IKA, Wilmington, NC, USA). The homogenized slurry was stirred with a mixture of NaCl solution (0.1 M) and toluene (9:1 *v*/*v*) to remove most lipids and proteins from the maize kernels. The mixture was stirred for 1 h and then allowed to stand until the starch granules settled at the bottom. The toluene layer and most of the NaCl solution layer, containing proteins and lipids, were removed. This procedure was performed several times until the toluene layer was clear. The remainder was repeatedly washed with water and pure ethanol to obtain starch granules, followed by air-drying at room temperature overnight.

### 2.3. Starch Extraction, Debranching, and Characterization by Size-Exclusion Chromatography (SEC)

The starch samples were extracted from the maize flour for SEC analysis of starch molecular structures following a method described elsewhere [12]. In brief, residual protein was removed from the flour samples using successive treatments with protease and sodium bisulfite solution followed by a centrifugation step. Most of the other nonstarch components were removed by dissolving the samples in DMSO, and the starch was then precipitated from the resulting soluble portion by centrifugation after adding sufficient ethanol. The extracted starch was debranched using isoamylase as described elsewhere [13]. The extracted whole and debranched starches were dissolved overnight at 80 ℃ in DMSO containing 0.5% (*w*/*w*) LiBr to yield a final concentration of 2 mg/mL and 4 mg/mL, respectively, prior to SEC analysis.

The debranched CLDs were characterized using an Agilent 1260 infinity SEC system (Agilent, Santa Clara, CA, USA) with a refractive index detector (Optilab UT-rEX, Wyatt, Santa Barbara, CA, USA) following a previously described method [14]. A combination of a GRAM precolumn, GRAM 100, and GRAM 3000 columns (Polymer Standard Service, Mainz, Germany) placed in a column oven at 80 °C were used for size separation. DMSO containing 0.5% (*w*/*w*) LiBr was used as eluent with a flow rate of 0.3 and 0.6 mL/min, respectively. Universal calibration was used to convert SEC elution time to the SEC separation parameter, the hydrodynamic radius *R*_h_ [14], using pullulan standards (Polymer Standards Services, Mainz, Germany) with a molecular weight range of 342–2.35 × 10^6^ Da. The SEC weight distribution obtained using a differential refractive index detector, *w*(log *R*_h_), gives (within arbitrary normalization) the total weight distribution (not molecular weight distribution) of molecules as a function of log *R*_h_ [14]. For the debranched samples, which are linear polymers, there is a unique relationship between size and molecular weight (which is not the case for branched polymers), and *R*_h_ can be converted to degree of polymerization (DP, being the number of monomer units in the chain) *X* using the Mark–Houwink equation [14].

### 2.4. Differential Scanning Calorimetry (DSC)

Thermal properties of purified starch granules from wet milling were measured by differential scanning calorimetry (DSC 200 F3, Netsch, Darmstadt, Germany). Starch (3 mg, dry weight basis) was precisely weighed into a crucible with 6 mg of water. The pans were sealed hermetically and allowed to equilibrate for 1 h at room temperature prior to the DSC measurement, followed by a holding step at 20 °C for 1 min. The scanning temperature range and heating rate were set at 20–120 °C and 10 °C min^−1^, respectively. An empty sealed pan served as the reference, and indium was used for calibration. Onset temperature (*T*_o_), peak temperature (*T*_p_), conclusion temperature (*T*_c_), melting temperature range (*T*_c_ − *T*_o_), and melting enthalpy (Δ*H*) were determined from each endotherm.

### 2.5. In Vitro Starch Digestion

The in vitro starch digestion procedure was carried out according to a previously described method [13]. Isolated starch granules (50 mg, dry weight basis) were firstly suspended in 2 mL of distilled water and gelatinized in a boiling water bath for 30 min with stirring and occasional vortex mixing. The gelatinized starch was stored at −20 ℃ overnight followed by freeze-drying. Each freeze-dried starch sample (retrograded starch) was dispersed at 37 °C in 7.5 mL of sodium acetate buffer (0.2 M, pH 6.0, containing 200 mM CaCl_2_, 0.49 mM MgCl_2_, and 0.02% NaN_3_). The starch suspension was added to an enzyme solution (0.5 mL) containing 12.5 μL of amyloglucosidase and 25 μg of pancreatin in sodium acetate buffer, and the mixture was incubated and stirred at 200 rpm in a water bath at 37 °C. Starch digesta (0.1 mL) were collected at 0, 10, 20, 40, 60, 90, 120, 180, 240, and 300 min, and the digestion was stopped by adding 0.9 mL of absolute ethanol. The digestion kinetics of retrograded starch (g/100 g of dry starch) were determined from the amount of glucose released in the supernatant, measured using the Megazyme D-Glucose (GOPOD Format) Assay Kit, with a conversion factor of 0.9 (the ratio of the molecular weight of the anhydroglucose monomer unit in starch to that of glucose). 

The digestogram was fitted using the logarithm of slopes (LOS) method [15,16]. This assumes that the digestion over a given time period obeys first-order kinetics for the fraction of undigested starch at time *t*, *C*(*t*):

C(*t*) = (1 − C_∞_) e^−kt^ + C_∞_ (1)

where *k* is the digestion rate coefficient and *C*_∞_ is the fraction remaining at long times. Application of the LOS method shows which steps of the digestion kinetics follow first-order kinetics [17]. The rate coefficient(s) (*k*) obtained by the LOS equation (there is more than one if the LOS analysis reveals more than one first-order region) were then used as an initial estimation for nonlinear least-squares fitting (NLLS) to obtain more accurate values of *k* and *C*_∞_ [17].

### 2.6. Fitting Amylose and Number Amylopectin CLDs

The CLDs in the amylose and amylopectin regions were fitted with the amylose model of Nada et al. [18] and the amylopectin model of Wu et al. [19]; see [5] for a recent review. The CLDs of amylopectin and amylose are separated by the value of DP where there is a clear change between the CLDs of the chains of amylopectin (which are short) and those of amylose (which are longer); this is at *X* ~ 100. The amylopectin model assumes that enzyme sets, each comprising one starch synthase (SS), starch branching enzyme (SBE), and starch debranching enzyme (DBE), operate concertedly to synthesize a component in the amylopectin CLD. The overall amylopectin CLD is the sum of the contributions of each enzyme set. The parameters in the model are, for the *i*^th^ amylopectin (Ap) enzyme set, *β*Ap,*i*, the ratio of the activity of SBE to that of SS (the activity of DBE is constrained by certain requirements and is implicit in those of SS and SBE), and *h*Ap,*i*, the relative activity of SS. For SEC CLD data (where some features of the fine structure are masked by band broadening), the amylopectin CLD can be fitted by three enzyme sets, denoted i, ii, and iii, and the relative contributions of enzyme set ii and iii to enzyme set i, *h*Ap,ii and *h*Ap,iii.

An amylose CLD usually presents distinct features (given good SEC separation) in the form of several maxima or shoulders. These features are associated with different enzyme sets, and the fitting parameters are *β*Am,*i* and *h*Am,*i*, with the same meaning as those for amylopectin [5].

### 2.7. Statistical Analysis

All measurements were performed in duplicate. All data are reported as mean ± standard deviation and analyzed by Minitab 16 (Minitab Inc, State College, PA, USA) with analysis of variation (ANOVA). The significant difference of the mean values was determined at *p* < 0.05.

## 3. Results

### 3.1. Chain-Length Distributions

**Figure 1:**
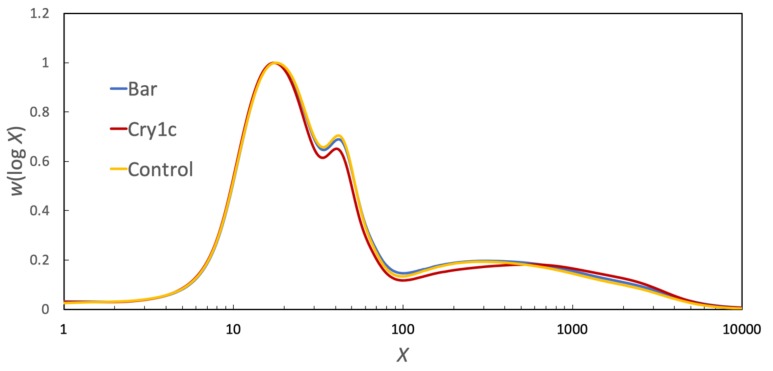
SEC weight distributions, *w* (log *X*), as functions of degree of polymerization (*X*) for debranched endosperm starch from different maize lines, normalized to the maximum height. Regions *i*, *ii*, an*d iii* are shown in the Appendix.

The weight CLDs of endosperm starch from the maize lines with different genetic background, as functions of DP *X*, are given in Figure 1. The CLDs were normalized to have the same global maximum. These show the well-known features of amylopectin and amylose molecules, with amylopectin chains having two peaks around DP 20 and 45, and amylose having one or more peaks around DP 300 and above. The CLDs for *Bar* lines and control lines were very similar. Although the amylose content of the three lines was not statistically different, the proportion of the chains with intermediate length (short amylose chains, DP 100~800) in *Cry1c* lines was lower than the others, which can also be observed in Figure 1. These differences will later be quantified using parameters obtained by fitting to the two biosynthesis models. Such distinct features in the molecular structure of starch can influence various properties of starch, as indeed will be seen.

### 3.2. Gelatinization Properties

The values of *T*_o_, *T*_p_, *T*_c_, (*T*_c_*-T*_o_), and Δ*H* of retrograded starches from the different lines are shown in Table 1. This shows a noticeable difference in the peak temperature of *Cry1c* samples compared with the others, which could be partly due to the influence of the difference in short (DP 6–12) amylopectin chains in *Cry1c* starch, as reflected in the different values of *β*Am,i (see below). The other thermal parameters from the two transgenic starches did not show significant differences.

### 3.3. In Vitro Digestibility

**Figure 2:**
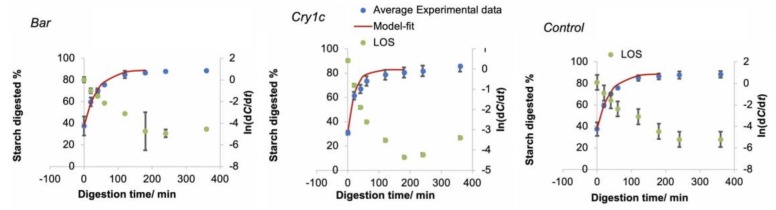
Digestograms of retrograded maize fitted using the nonlinear least-squares fit (NLLS) and LOS treatment [ln(d*C*/d*t*) against *t*] based on duplicate measurements. The NLLS treatment is only used over the region in which the LOS plots suggest a single first-order loss.

As shown in Figure 2, the LOS data treatment shows an acceptable linearity of the digestograms up to ~200 min. The digestion data over this range were fitted with a single first-order loss step using the NLLS method, to yield values of k and C∞. The digestograms over the whole time range calculated using these parameters are also shown. As shown in Table 2, significant differences of digestion rate coefficient *k* and the fraction remaining after long-time hydrolysis, *C*∞, were found between *Cry1*c and the other two lines; the analysis used two bioreplicas for each transgenic line and each replica had two technical repeats.

### 3.4. Parameterizing Amylopectin and Amylose CLD with Biosynthesis Models

Details of the various components in the fitting process are given in the Supporting Information, Figure S1; there are several examples of such fitting given in more detail in recent literature (e.g., [5]). The amylopectin and amylose CLD fitted parameters are given in Table 3. All the parameters are biologically meaningful. The parameters, represented in Table 3, are the ith amylopectin (Ap) enzyme set, βAp,i, the ratio of the activity of SBE to that of SS, and hAp,i, the relative activity of SS. There are three enzyme sets, denoted i, ii, and iii, fitting the amylopectin CLD, and the parameters are the ratio of enzyme set ii and iii to set i, denoted hAp,ii and hAp,iii. The hAp,ii values of *Cry1c* were significantly lower (*p* < 0.05) than the others, while the βAp,i values of *Cry1c* were significantly higher (*p* < 0.05). A higher value of βAp,i in *Cry1c* lines means a higher ratio of the activity of SBE to that of SS in *Bar* and *Control* lines, which is related to the synthesis of the single-lamellae spanning chains (DP 6–33), dominated by enzyme set i. No significant difference was observed in the *β*Ap,iii values of any of the transgenic lines and control lines, which shared a similar ratio of enzyme set iii activity, which dominates synthesis of the two-lamellae spanning chains (DP 34−67). The parameter *h*Ap,*i* is the relative contribution of enzyme set *i* to the entire amylopectin CLD. The difference for enzyme set i was more significant than that of enzyme set iii, indicating that the proportion of long amylopectin chains in *Cry1c* samples was lower than the others, due to the distinctly different activities of enzyme set i. The lower contribution of enzyme set iii (lower value of *h*Ap,iii) was observed in *Cry1c* lines compared with the others. 

The amylose CLD data were best fitted with three distinct enzyme environments (regions). The parameterization results are given in Table 3. There was a significant difference in the SBE/GBSS activity ratio *β*Am,i for *Cry1c* and the other two lines, which is for amylose region i (DP 10−350). 

The amylopectin and amylose models give independent starch structural parameters correlating with the in vitro digestion rate. The amylose content and amylopectin CLD fitted parameters showed no significant difference between the samples; for amylose, the *β*Am,i values of region 1 and region 2 correlated negatively with the digestion rate coefficient. 

## 4. Discussion

The objective in this paper was to see if there are variations in structurally related functional properties such as digestibility and thermal properties between the transgenic lines and control lines. Certain structural changes are known to affect some functional properties, for reasons discussed in the literature (e.g., [20,21,22,23]) (it is noted that the present sample set is too small for meaningful discussion of the mechanisms of these structure–property relations). The effects of amylose and amylopectin structures (chain-length distributions) are here related to thermal properties and in vitro digestion rates. It has been established [24] that other structural features, such as the size distributions of the whole (undebranched) starch molecules, do not affect these properties. The CLDs are fitted to biosynthesis models which encapsulate these distributions in terms of a small number of biologically meaningful parameters, which are used to find correlations with starch enzyme activities.

The pest-resistant gene (*Cry1c* gene) and herbicide-resistant gene (*Bar* gene), both of which are unrelated to genes controlling starch synthesis [25,26,27], are exogenous genetic fragments used in crop improvement. In plants with these genes, statistically significant differences (although always being aware of the limitations of p values [28]) were found in starch structure and in two of the properties which depend on these structural features: the retrograded starches of *Cry1c* lines have lower gelatinization temperature and faster digestion rate.

Since the samples were all cooked, and thus could be digested quite quickly by α-amylase, some of the starch would have been degraded into reducing sugars during the sampling process, which is why starch digestion did not start at 0% in the digestogram. This phenomenon is commonly found among cooked starchy food digestion measurements [29]. The LOS analysis indicated that the first 200 min of the digestion process followed first-order kinetics, while the digestion rate slowed down at longer times, leading to the rapid increase at the last time point (360 min), as the digestible starch was essentially completely depleted. This shortcoming of the LOS method is not found in the NLLS method [17]. The more abundant short amylopectin chains of *Cry1c* were one of the factors accelerating the digestion rate [23]. The structural differences were parameterized by fitting to biosynthesis models, whose parameters are the relative activities and contributions of starch synthesis enzymes. As seen in Table 3, a higher ratio of the activity of SBE to that of SS was observed in *Cry1c* lines during amylopectin synthesis, since the values of βAp,ii and βAp,iii were both higher than in the other lines. SBEII is able to transfer shorter chains (DP ≤ 12) than SBEI, which participates in amylopectin synthesis rather than amylose synthesis [30]. SBEIIb is specific for maize endosperm starch synthesis [31]. The higher activity of SBEIIb, which is inferred from the model fitting, implied a higher amount of short amylopectin and amylose chains in *Cry1c* lines. The changes in structural features also could have arisen from lower SS activity. There are three types of SSs with multiple isoforms. SSs are responsible for elongating (1→4)-α-glucan chains of amylose and amylopectin. The shortest chains of amylopectin are the preferred substrates for SSI, while SSII is believed to be responsible for intermediate-length chain synthesis (DP 12–24). SSIII is most abundant in tubers, and responsible for long amylopectin chains (DP > 30). The CLD fitting parameters quantify the starch CLDs, with the parameters from the model fitting being enzyme activity ratios and thus reflecting genetic differences. The altered starch enzyme activity could be the consequence of somaclonal variations (genetic variation in plants generated from somatic cells cultured in vitro), triggered by the callus regeneration [32]. 

The present study examined starch molecular structure, thermal properties, and digestibility of retrograded starch from *Bar* and *Cry1c* transformed lines. No significant difference was found in amylose content, but the *Cry1c* lines had noticeably different molecular structures from the *Bar* and control lines: fewer short amylose chains and more long amylopectin chains. This is similar to a previous study [9] showing that the existence of *Cry1ab*, regulated by *Ubi* promoter, might influence starch properties, including reduced viscosity; we suggest here that this is due to the type of pleiotropic effects on starch molecular structures.

Genetic modification in non-starch-related genes has been shown here to be able to lead to alterations in starch structures and properties, in particular, the gelatinization and digestibility properties studied here. In this case, the starch harvested from *Cry1c* lines, in terms of nutritional value, may be less desirable than the *Bar* and untransformed lines, since higher starch digestibility is associated with type II diabetes, colorectal cancers, and obesity. This manuscript focuses on seeing if there can be alterations in starch structure among transgenic maize lines where the transgenes are not supposed to affect starch biosynthesis. Because a change is seen in starch structure, it shows that it is possible to have deleterious effects on starch structure where the genetic alterations should have had no such effect. Seeing if the transgene insertion is close to genes involved in starch biosynthesis would help understand the mechanism for the observation and point the way to avoiding the problem in the future. The likelihood of pleiotropic effects on starch molecular structure and properties should be considered along with crop improvement when creating transformed plants. The observation that commonly used transformations, which were not expected to have any effect on starch structure, in fact in one case had a significant effect, fulfills the aim of this paper. The mechanism whereby this occurred is of interest, and merits future investigation.

## Figures and Tables

**Table 1:** Thermal properties of retrograded starches of transgenic lines and control lines from DSC. Mean ± SD is calculated from duplicate measurements. Different letters within a row indicate significant differences (*p* < 0.05).

Properties	*Bar*	*Cry1c*	Control
T_o_ (°C)	43 ± 1 a	43.6 ± 0.4 a	44 ± 1 a
T_p_ (°C)	54 ± 1 a	51.1 ± 0.3 b	53 ± 1 a
T_c_ (°C)	65 ± 2a	64.3 ± 0.2 a	64.6 ± 0.2 a
T_c_-T_o_ (°C)	26 ± 2 a	21 ± 1 a	27 ± 1 a
∆H (J g−1)	3.6 ± 0.8 a	3.0 ± 0.1 a	3.7 ± 0.5 a

**Table 2:** Starch digestion rate coefficient and percentage of undigested starch for each phase, obtained by NLLS. Mean ± SD is calculated from duplicate measurements. Different letters within a row indicate significant differences (*p* < 0.05).

Digestion Parameter	*Bar*	*Cry1c*	*Control*
*k*/min^–1^	0.025 ± 0.001 a	0.043 ± 0.000 b	0.027 ± 0.005 a
*C*_∞_/%	10 ± 1 a	17 ± 4 b	11 ± 3 a

**Table 3:** Fitted molecular structural parameters of starch in the retrograded maize starch samples, made from duplicate measurements. Different letters within a row indicate significant differences (*p* < 0.05).

-	Amylose Content (%)	*β*Am,i/10^−3^	*β*Am,ii/10^−3^	*β*Am,iii/10^−3^	*h*Am,ii	*h*Am,iii	*h*Ap,iii	*h*Ap,ii	*β*Ap,ii/10^−2^	*β*Ap,iii*_/_*10^−2^
*Bar*	26 ± 1 a	8.8 ± 0.2 a	2.8 ± 0.1 a	1.2 ± 0.1 ab	10.6 ± 0.1 a	13.6 ± 0.1 a	1.01 ± 0.01 a	0.35 ± 0.01 a	3.89 ± 0.04 a	4.6 ± 0.2 a
*Cry1c*	25 ± 1 a	6.6 ± 0.1 b	2.5 ± 0.1 b	1.2 ± 0.03 b	11.3 ± 0.1 b	14.2 ± 0.1 b	1.02 ± 0.00 b	0.32 ± 0.01 b	4.1 ± 0.1 b	4.6 ± 0.2 a
*Control*	25.3 ± 0.3 a	9.1 ± 0.1 a	3.1 ± 0.2 c	1.5 ± 0.1a	10.4 ± 0.1 a	13.4 ± 0.2 a	1.02 ± 0.01 b	0.34 ± 0.01 ab	3.8 ± 0.1 a	4.4 ± 0.03 a

## Supplementary Materials

The following are available online at https://www.mdpi.com/2304-8158/9/2/222/s1, Figure S1. Fitting experimental amylose CLDs to the biosynthesis model, showing the experimental, final fitted, and the broadened components for each region. For all samples, the parameters giving the minimum chain lengths for action of the isoforms of SBE in amylopectin were chosen to be X0(i) and X0(iii) as 8 and 3, respectively, while Xmin(i) and Xmin(iii) were set as 9 and 8−9. SBE snips off a piece from a chain with more than a minimal number of monomer units, X0, and the snipped piece must be more than a different minimal number of monomer units, Xmin-see Wu, A.C., Gilbert, R.G., 2010; Biomacromolecules 11, 3539−47.

## Abbreviations

CLD: chain-length distribution; SEC, size-exclusion chromatography; DMSO, dimethyl sulfoxide; DP, degree of polymerization; DSC, differential scanning calorimetry; LOS, logarithm of slopes.
