# Supplementary material for: Effects of Nonstarch Genetic Modifications on Starch Structure and Properties
Source: Foods. 2020 Feb 20;9(2):222. doi: 10.3390/foods9020222 (PMC7074516; doi:10.3390/foods9020222)
Supplement: Supplementary file 1 [file foods-09-00222-s001.pdf]

Supporting Information

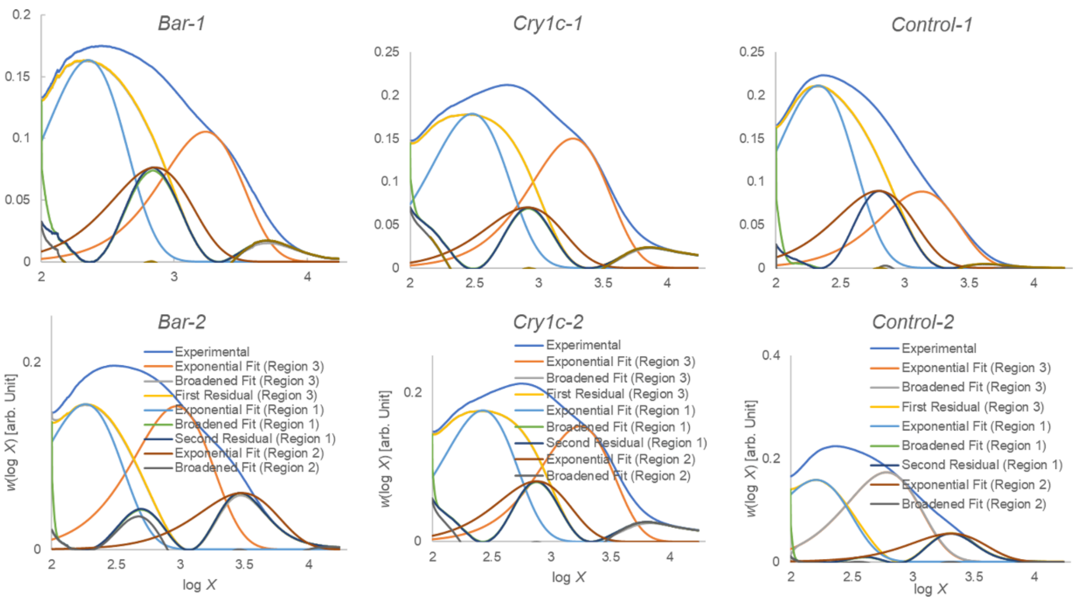

Figure A1. Fitting experimental amylose CLDs to the biosynthesis model, showing the experimental, final fitted, and the broadened components for each region. For all samples, the parameters giving the minimum chain lengths for action of the isoforms of SBE in amylopectin were chosen to be $X_{0(\text{i})}$ and $X_{0(\text{iii})}$ as 8 and 3, respectively, while $X_{\text{min(i)}}$ and $X_{\text{min(iii)}}$ were set as 9 and 8-9. SBE snips off a piece from a chain with more than a minimal number of monomer units, X_0, and the snipped piece must be more than a different minimal number of monomer units, X_{min} – see Wu, A.C., Gilbert, R.G., 2010; *Biomacromolecules* 11, 3539-47.
